# Supplementary material for: Predictors of Burnout and Well-Being Among Veterinarians in Slovenia
Source: Vet Sci. 2025 Apr 20;12(4):387. doi: 10.3390/vetsci12040387 (PMC12030772; doi:10.3390/vetsci12040387)
Supplement: Supplementary file 1 [file vetsci-12-00387-s001.zip › vetsci-3536103-supplementary.pdf]

Table 1S: Mental and Physical Health Challenges Among Veterinarians

	N	%	Valid %
Have you felt burnt out by your work in the past month?	208	44,0	52,8
Have you been concerned in the past month that your work is making you emotionally numb?	187	39,5	47,5
Have you struggled with feelings of sadness, depression, or hopelessness in the past month?	163	34,5	41,4
Have you fallen asleep while driving or waiting in traffic in the past month?	60	12,7	15,2
Have you felt overwhelmed by the number of tasks piling up to the point of losing control in the past month?	176	37,2	44,7
Has your emotional well-being (such as anxiety, depression, or irritability) been a concern for you in the past month?	198	41,9	50,3
Has your physical health limited your ability to complete daily tasks at home or outside the home in the past month?	144	30,4	36,5
Missing data	79	16,7	
Total	473		

Table 2S: Burnout Levels Among Respondents

	N	%	Valid %
Low burnout (0–2 symptoms)	179	37,9	45,5
Moderate burnout (3–4 symptoms)	104	22,0	26,4
High burnout (5–7 symptoms)	112	23,7	28,3
Missing data	79	16,7	
Total	473	100,0	

Table 3S: Distribution of respondents by year of graduation compared to population data

	N	%	Valid %	Population data
1974 - 1984	33	7,0	7,2	4,9
1985 - 1994	86	18,2	18,7	18,6
1995 - 2004	132	28	28,7	23,4
2005 - 2014	128	27,1	27,8	26,8
2015 - 2023	81	17,1	17,6	26,3
Missing data	13	2,7		
Total	473	100		

Table 4S: Gender distribution of respondents

	N	%	Valid %
Female	247	52,2	63,2
Male	137	29	35
Unspecified	7	1,5	1,8
Missing data	82	17,3	
Total	473	100	100

Table 5S: Number of household members

	N	%	Valid %
1 person	-	-	-
2 persons	94	19,9	24,2
3 persons	83	17,5	21,4
4 persons	105	22,2	27,1
5 or more persons	59	12,4	15,2
Missing data	95	20,1	-
Total	473	100	100

Table 6S: Respondents living with children

	N	%	Valid %
Yes	243	51,4	63,4
No	140	29,6	36,6
Missing data	90	19	-
Total	473	100	100

Table 7S: Number of children in the household

	N	%	Valid %
1 child	85	18	35,3
2 children	103	21,8	42,7
3 or more children	51	10,8	21,1
Total	243	51,4	100

Table 8S: Financial self-assessment of living conditions

	N	%	Valid %
Living very well on current income	32	6,8	8,2
Living comfortably on current income	228	48,2	58,5
Just managing on current income	111	23,5	28,5
Difficult to live on current income	16	3,4	4,1
xtremely difficult to live on current income	3	0,6	0,8
Missing data	83	17,5	
Total	473	100	

Table 9S: Childhood environment of respondents

	N	%	Valid %
Large city	53	11,2	13,6
Suburb	37	7,8	9,5
Small town	123	26	31,5
Village	98	20,7	25,1
Farm/rural house	80	16,9	20,5
Missing data	82	17,3	-
Total	473	100	100

Table 10S: Job Position of Respondents

	N	%	Valid %
Managerial position (director, dean, head of department, senior professional lead)	96	20,3	22,8
Ownership position (owner, co-owner)	70	14,8	16,6
Employed without a managerial or ownership position	285	60,3	67,7
Missing data	52	11,0	
Total	473		

Table 11S: Employment Status of Respondents

	N	%	Valid %
Employed in veterinary practice (clinic)	291	61,5	62,2
Employed in education, research, or diagnostics	71	15,0	15,2
Employed in the industry	18	3,8	3,9
Employed in public administration	76	16,1	16,2
Employed outside the veterinary profession	13	2,7	2,8
Unemployed	3	0,6	0,6
Other	19	4,0	4,0
Missing data	5	1,1	1,1
Total	473		
